# *MrERF*, *MrbZIP*, and *MrSURNod* of *Medicago ruthenica* Are Involved in Plant Growth and Abiotic Stress Response

**DOI:** 10.3389/fpls.2022.907674

**Published:** 2022-06-02

**Authors:** Rina Wu, Bo Xu, Fengling Shi

**Affiliations:** Key Laboratory of Grassland Resources of the Ministry of Education, College of Grassland Resources and Environment, Inner Mongolia Agricultural University, Hohhot, China

**Keywords:** *Medicago ruthenica*, abiotic stress, plant growth, transgenic tobacco, morphology, physiology

## Abstract

Abiotic stresses affect plant growth and productivity. The outstanding stress resistance of *Medicago ruthenica* makes it a desirable gene resource to improve the stress tolerance of other plants. The roles of three differently expressed genes [(DEGs) (*MrERF*, *MrbZIP*, and *MrSURNod*)] from *M. ruthenica* in stress resistance have not been fully elucidated. Therefore, we constructed their expression vectors, transformed them into tobacco, and subjected transgenic lines to abiotic stresses. Through comprehensive bioinformatics, transcriptomic, morphological, and physiological analyses of transgenic lines, we have revealed the critical role of these three DEGs in plant growth and abiotic stress response. The upregulation of genes enhanced the germination rate, biomass, root length number, etc. Additionally, the accumulation of osmolytes increased the activity of antioxidant enzymes. These genes are also associated with improved seed yield, increased branching, and early flowering, thereby shortening the growth period. Potentially, this is one of the ways for tobacco to cope with stress. Furthermore, the resistance of transgenic tobacco expressing *MrERF* or *MrbZIP* was better than that with *MrSURNod*. *MrERF* and *MrbZIP* can improve drought and salt tolerance of plants, whereas *MrSURNod* is beneficial in improving drought and cold resistance. Moreover, *MrERF* or *MrbZIP* can promote root elongation and increase the root number, whereas *MrSURNod* mainly promotes root elongation. This may be the reason why stress resistance conferred by *MrSURNod* is weaker than that associated with the other two genes. Overall, *MrERF*, *MrbZIP*, and *MrSURNod* positively modulate plant growth and stress tolerance.

## Introduction

Grassland ecological environment is extremely complex, and forages are often exposed to cold, arid, and other stress environments not conducive to plant growth and high yield. Hence, it is necessary to develop new forage varieties with strong tolerance and high yield and protein. With the development of molecular technologies, scientists use genetic engineering to transfer stress-resistance genes to plants with a high yield but weak resistance to improve their stress resistance, which has become a new direction for plant resistance research.

*Medicago ruthenica*, a forage legume, is widely distributed in alpine and desertification grasslands in northern China, having the advantages of cold, drought, salinity, barren, and trampling resistance. Therefore, this species can grow well in cold and arid areas (Yang et al., 2011) and has tremendous potential for grassland improvement, ecological management, and grassland industry development (Zhao et al., 2013). Although it belongs to the genus *Medicago* as well as alfalfa (*Medicago sativa*), it has higher nutrient-use efficiency than alfalfa (Campbell et al., 1997) and can be used as a high-quality protein feed. In addition, domestic and foreign scholars agree that its outstanding resistance to drought, cold, salinity, and alkalinity stresses makes it not only a high-quality parent in breeding, but it can also be used as a gene resource to improve the stress resistance of alfalfa and other forages. It is thought that the new varieties of alfalfa with high yield and high stress resistance can be obtained by transferring the genes from *M. ruthenica* to alfalfa using transgenic technology (Campbell, 2003). Therefore, on the basis of previous morphological and physiological studies, transcriptome sequencing of *M. ruthenica* under drought was carried out to screen drought resistance-related genes.

In order to cope with various environmental stresses, plants have evolved a series of regulatory mechanisms. Plants perceive different stress signals from the outside and respond accordingly, and various hormonal and metabolic signaling pathways are involved in the processes. Notably, plant hormone signal transduction pathway plays a key role in resisting abiotic stress (Depuydt and Hardtke, 2011). Among them, ABA regulates not only plant growth and development but also plays an important role in plant responses to abiotic stress (Gupta and Rashotte, 2014; Vishwakarma et al., 2017). Numerous studies have shown that bZIP and AP2/EREBP transcription factors are involved in ABA signal transduction and stress responses in plants. Here, we studied three DEGs from *M. ruthenica* identified under drought, including two transcription factors (*MrERF*, *MrbZIP*) and one unknown gene (*MrSURNod*). *MrERF* belongs to AP2/EREBP family and *MrbZIP* to bZIP family. It is well documented that bZIP family genes are involved in plant adaptation to drought stress by regulating the expression of drought resistance-related genes (Wang et al., 2017). For example, overexpression of *SlbZIP1* (Zhu et al., 2018), *AtABF3* (Wang et al., 2016) or *CaDILZ1* (Lim et al., 2018) enhanced drought tolerance of transgenic lines. However, they can also have an opposite effect, e.g., *SlbZIP38* in tomato, enhancing plant sensitivity to drought (Pan et al., 2017). AP2/EREBP is involved in regulating abiotic stress responses as well as plant hormones (e.g., ABA) (Dossa et al., 2016). Studies have proved that overexpression of ERF in rice, tobacco, and other plants can improve plant tolerance to drought or high salinity. For example, the overexpression of *OsERF48* (Jung et al., 2017), *OsERF71* (Lee et al., 2016), and *PpERF023* (Bielsa et al., 2018) enhanced drought resistance of transgenic plants. The overexpression of *Arabidopsis ERF1* (Cheng et al., 2013) and wheat *TaERF3* (Rong et al., 2014) improved the drought and salt tolerance of transgenic lines. The overexpression of *MfERF1* in alfalfa improved the cold tolerance of transformed plants (Zhuo et al., 2018), and *ERF105* (Bolt et al., 2017), *ERF102*, and *ERF103* (Illgen et al., 2020) were confirmed to play important roles in cold acclimation of *Arabidopsis thaliana*.

In addition to *bZIP* and *AP2/EREBP*, an unknown gene *MrSURNod* with a high homology to *Medicago truncatula* was also identified in our study. The above genes were up-regulated under drought stress and may play an important role in the adaptation of *M. ruthenica* to drought. Therefore, the transcription factors *MrbZIP* and *MrERF* involved in ABA signal transduction pathways and an unknown gene *MrSURNod* were selected to construct plant expression vectors and transform them into tobacco. Three resistant tobacco plants were treated with drought, low temperature, and NaCl, and the expression pattern of the genes under stress was detected by qRT-PCR. We characterized the differences in phenotype and physiological and biochemical indices between WT and over-expression plants. Our research put forward new ideas for improving the abiotic stress of plant and laid the foundation for breeding forages for stress resistance.

## Materials and Methods

### Plant Materials and Growth Conditions

*M. ruthenica* (L.) cv. Zhilixing was selected as the experimental material. The seeds of this variety were collected in September 2008 from the experimental fields of Inner Mongolia Agricultural University located in Hohhot, Inner Mongolia, China. All seeds were soaked in concentrated H_2_SO_4_ for 5–8 min in order to break the hard coat and were then germinated on 1/2 MS medium until the seedlings grew 6–8 leaves. Subsequently, leaf samples were snap-frozen in liquid nitrogen and stored at −80°C for RNA extraction. Tobacco (*Nicotiana benthamiana*) was used for the generation of overexpression transgenic plants. Tobacco seeds came from the seed storage of Inner Mongolia Agricultural University. *M. ruthenica* and tobacco plants were grown in a greenhouse under controlled conditions (25^°^C, 16-h light/8-h dark, 50% humidity).

### Gene Cloning, Sequencing, and Bioinformatics Analysis

Total RNA was extracted using the TRIzol reagent (Invitrogen, Carlsbad, CA, United States) following the manufacturer’s protocol. A UEIris II RT-PCR System for first-strand cDNA synthesis (with dsDNase) (US Everbright, Suzhou, China) was used to synthesize cDNA. Polymerase chain reaction (PCR) was performed according to the manufacturer’s recommended protocol. After detection by agarose gel electrophoresis, the PCR product bands were recovered using a gel recovery kit (Omega, United States) and sequenced. Bioinformatics analysis was conducted through the following websites: ExPASy tools,^1^ Wolfpsort,^2^ TMHMM Server v.2.0,^3^ and CDD.^4^ The phylogenetic tree was constructed through neighbor-joining analysis using MEGA 7.0 (bootstrap 1000).

### Plasmid Construction and Plant Transformation

The CDSs of *MrERF*, *MrbZIP*, and *MrSURNod* were ligated into the pCAMBIA2300-GFP vector using a homologous recombination system. The primer sequences used for vector construction are listed in Supplementary Table 1. The recombinant pCAMBIA2300—*MrERF*—GFP, pCAM BIA2300—*MrbZIP*—GFP, pCAMBIA2300—*MrSURNod*—GFP, and pCAMBIA2300—GFP plasmids were transformed into *Agrobacterium tumefaciens* (GV3101) for plant transformation. A stable transformation of tobacco was performed using a leaf disk co-cultivation protocol. The positive transgenic tobacco lines were selected by 100 mg/L kanamycin and confirmed by genomic DNA PCR (Plant Genome DNA Extraction and Amplification Kit (Biomed, Beijing, China), following the manufacturer’s protocol). After full development, the gene expression in the positive transgenic tobacco leaves was confirmed by qRT-PCR. The leaves with successful expression were selected for subculturing until roots formed, and seedlings were then transferred to soil for subsequent study.

### Expression Pattern of *MrbZIP*, *MrERF*, and *MrSURNod*

The methods of RNA extraction and cDNA synthesis were described earlier. The qRT-PCR assays were performed using Fast Super EvaGreen^®^ qPCR Master Mix (US Everbright, Suzhou, China) in an ABI 7500 system (Applied Biosystems, United States) with the following program: 95^°^C for 2 min; 45 cycles of 95^°^C for 10 s, 55^°^C for 10 s, 72^°^C for 30 s, 95^°^C for 15 s, and 60^°^C for 60 s. The primers used for the amplification of *MrERF*, *MrbZIP*, and *MrSURNod* (Supplementary Table 2) were designed according to the CDS of *MrERF* (Cluster-20905.1), *MrbZIP* (Cluster-60183.76174), and *MrSURNod* (Cluster-60183.75597). All the primers were synthesized by Invitrogen (Beijing, China). A 20 μL reaction mix was set up containing 10 μL of 2 × Fast Super EvaGreen^®^ Master Mix, 1 μL of 10 × ROX, 5.5 μL of ddH_2_O, 3 μL of cDNA, and 0.5 μg of each primer. The relative expression changes of the endogenous reference and tested genes were analyzed by the 2^–ΔΔ*CT*^ method.

### Abiotic Stress Treatment

#### The Resistance of Seeds at Germination Stage

Drought and salt treatment: The seeds of the transgenic (*MrERF*, *MrbZIP*, and *MrSURNod*) and WT lines were germinated on 1/2 MS medium containing mannitol (100, 200, and 300 mM) or NaCl (100, 150, and 200 mM) (Petri dish diameter 10 cm). Medium without additives was used as control (25°C, 16-h light/8-h dark, 50% humidity). Cold stress: The seeds were placed on 1/2 MS medium and placed in an incubator at 4°C. Three biological replicates were included in each treatment. Two weeks later, we scored the seed germination rate of transgenic and WT tobacco lines under different conditions.

#### The Resistance Identification at Seedling Stage

Seedlings were grown in pots (diameter 58 mm, depth 110 mm), and the planting substrate was organic nutrient soil that was purchased from Zhongshun Garden Machinery Co., Ltd. For the drought treatment, 5-week-old transgenic tobacco lines were subjected to no watering for 9 days. For the NaCl stress treatment, 5-week-old plants of the *MrERF*, *MrbZIP*, *MrSURNod*, and WT tobacco lines were watered with 200 mM NaCl for 9 days. For cold stress, seedlings were moved into an incubator set at 4°C and 16-h light/8-h dark. Leaves from tobacco lines were collected after 3, 6, and 9 days of stress. Three replicates per treatment were tested. All samples were collected at 10:00 am, snap-frozen in liquid nitrogen, and stored at −80°C. The gene expression of *MrERF*, *MrbZIP*, and *MrSURNod* in treated seedlings was detected after 9 days of growth under stress. Root length and number, plant height, and biomass at different time points during treatment were recorded. Soluble sugars, proline, MDA content, and SOD and POD under the treatment of drought, NaCl, and cold were measured. In addition, seed yield per plant was recorded.

#### Statistical Analysis

Statistical analyses were performed using SPSS Statistics (version 21, IBM, Chicago, IL, United States) Excel 2016 (Microsoft, Washington, DC, United States). Three biological replicates were included in each experiment. The data are presented as the means ± SDs (*n* = 3). The differences among the tested lines were assessed using the Duncan multiple comparison test. The level of significance was set at *p* < 0.05.

## Results

### Bioinformatics Analysis of *MrERF*, *MrbZIP*, and *MrSURNod*

**FIGURE 1:**
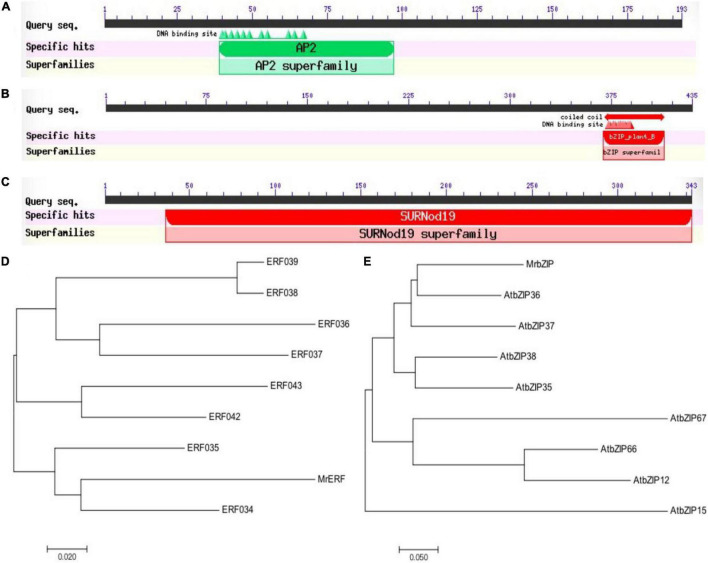
Prediction of the conserved domains, phylogenetic tree. **(A)** Conserved domain of *MrERF*; **(B)** conserved domain of *MrbZIP*; **(C)** conserved domain of *MrSURNod*; **(D)** phylogenetic tree of *MrERF*; and **(E)** phylogenetic tree of *MrbZIP*.

The fragments of *MrERF*, *MrbZIP*, and *MrSURNod* were amplified from *M. ruthenica* by RT-PCR. The coding regions (582, 1,308, and 1,029 bp) were cloned, sequenced, and submitted to the GenBank (accession numbers: MW811334, MW811335, and MW811336). The deduced amino acid sequences were 150 (*MrERF*), 427 (*MrbZIP*), and 340 amino acids (*MrSURNod*) (Supplementary Figure 1). The *MrbZIP* deduced protein has a bZIP domain of 54 amino acids comprising a leucine zipper. *MrERF* encodes a conserved AP2 domain (Figure 1). They have no transmembrane regions, whereas *MrSURNod* contained one SURNod domain and transmembrane regions (Supplementary Figure 2). Subsequently, phylogenetic trees were constructed using MEGA (version 7.0, Mega Limited, Auckland, New Zealand) (Figure 1). The amino acid sequences of *MrERF* and *MrbZIP* showed the highest similarity with the protein sequences from *Arabidopsis thaliana* (*AtERF034* and *AtbZIP36*). The unknown gene *MrSURNod* had less than 40% homology to other sequences. The subcellular localization analysis implied the possible localization of *MrERF* and *MrbZIP* in the nucleus and *MrSURNod* in the chloroplast (Supplementary Figure 3). These findings demonstrated that *MrERF*, *MrbZIP*, and *MrSURNod* were sensitive to drought and were rapidly up-regulated, suggesting the roles of the ERF, bZIP, and MrSUDNod proteins in responses to drought.

### Vector Construction and Genetic Transformation

**FIGURE 2:**
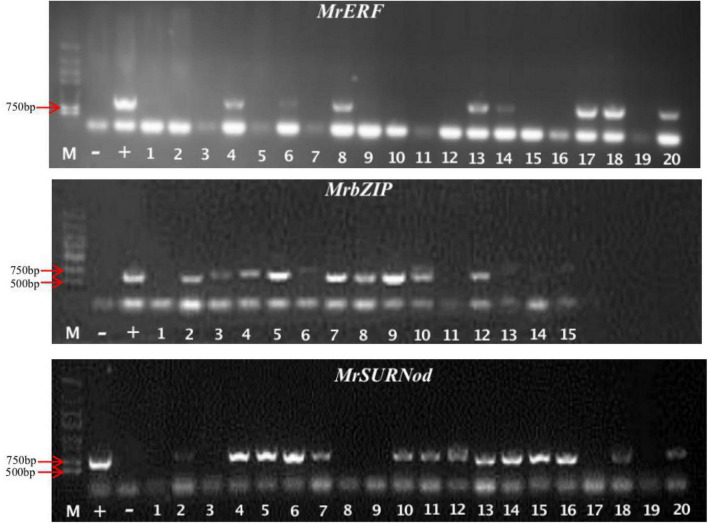
Identification of tobacco transgenic lines by PCR. “M”, Marker (DL2000); “–”, negative control; “+”, positive control, the rest were transgenic plants.

The target gene fragments of *MrbZIP*, *MrERF*, and *MrSURNod* were successfully ligated into the plant expression vector pCAMBIA2300-GFP, denoted as pCAMBIA2300-76174-GFP, pCAMBIA2300-20905.1-GFP, and pCAMBIA2300-75597-GFP, respectively (Supplementary Figures 4–6). Subsequently, the recombinant plasmids were transformed into agrobacterium. The results of colony PCR showed that the three vectors had been successfully transformed into agrobacterium strains and could be used later for plant transformation (Supplementary Figure 7). To investigate the biological functions of the three genes in response to abiotic stresses, tobacco (*Nicotiana tabacum*) leaves were transformed with above-mentioned fusion constructs *via* Agrobacterium-mediated transformation. Kanamycin-resistant T0 plants were evaluated with RT-PCR. The result showed that all transgenic plants had acquired the PCR product that was not present in WT (Figure 2). Positive transgenic plants containing *MrbZIP*, *MrERF*, or *MrSURNod* were cultured in water and transplanted to the soil a week later. Finally, the seeds of T0 generation were harvested.

### Identification of Transgenic Tobacco Plants

**FIGURE 3:**
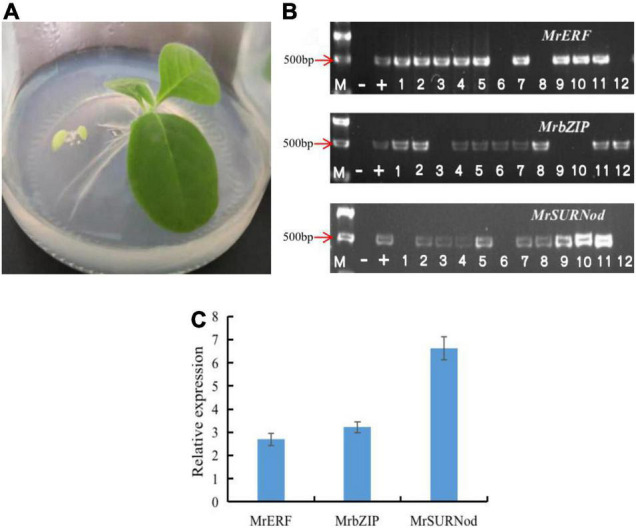
Transgenic plant identification. **(A)** Antibiotic selection; **(B)** identification of transgenic plants by PCR [“M”, Marker (DL2000); “–”, negative control; “+”, positive control, the rest were transgenic plants]; **(C)** qRT-PCR analysis of *MrERF*, *MrbZIP*, and *MrSURNod* in transgenic tobaccos.

The T0 generation tobacco seeds were screened on 1/2 MS medium containing 100 mg/L kanamycin (Figure 3A). The genomic DNA of transgenic plant leaves was extracted from healthy young leaves for PCR detection (Figure 3B). The results preliminarily proved that exogenous genes (*MrERF*, *MrbZIP*, and *MrSURNod*) had been successfully integrated into the tobacco genome. To detect the transcription expression of the genes, the transgenic plants with positive PCR detection were selected, and RNA was extracted. The results showed that exogenous genes in some transgenic plants were expressed, but the expression levels differed. After antibiotic screening, PCR detection, and qRT-PCR, T1 transgenic tobacco plants with stable integration of exogenous genes and successful expression were obtained (Figure 3C), including 68 plants with *MrERF*, 71 with *MrbZIP*, and 66 with *MrSURNod*; they were used for subsequent molecular, morphological and physiological stress resistance studies.

### Overexpression of *MrERF*, *MrbZIP*, and *MrSURNod* Affects Plant Growth and Development

**FIGURE 4:**
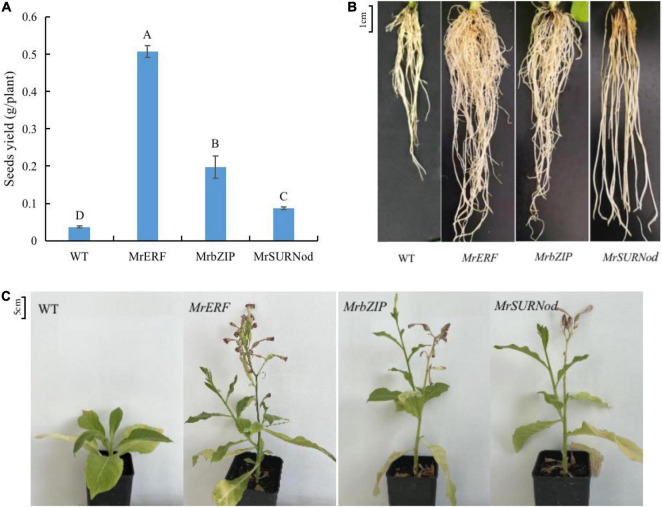
Effects of *MrERF*, *MrbZIP*, and *MrSURNod* on plant growth. **(A)** Seed yield of different tobacco lines (Different capital letters in the figure indicate significant differences at 0.05 level among different transgenic treatments). **(B)** Root morphology of different tobacco lines. **(C)** Morphological characteristics of aboveground parts of different tobacco lines.

To reveal whether the overexpression of *MrERF*, *MrbZIP*, or *MrSURNod* affects the flowering and fruiting of transgenic tobacco, the phenotypic characteristics at the mature stage under normal growth conditions were observed. The results showed that the over-expression transgenic lines grew better than WT, showing greater height, more branches, and earlier flowering (Figure 4C). In addition, the seed yield was significantly higher in transgenic tobacco than in WT (Figure 4A), being 14. 25-, 5. 56-, and 2.44-fold that of WT for *MrERF*, *MrbZIP*, and *MrSURNod* lines, respectively. The seed yield of the transgenic line (with *MrERF*) was significantly higher than others (*P* < 0.05). Transgenic plants also had longer and more numerous roots than WT (Figure 4B). This may contribute in improving the plant resistance. These results suggest that *MrERF*, *MrbZIP*, and *MrSURNod* are involved in plant growth. Their overexpression in tobacco-induced plant branching and earlier flowering and fruiting, thereby shortening the growth period.

### Expression of *MrERF*, *MrbZIP*, and *MrSURNod* Under Abiotic Stress

**FIGURE 5:**
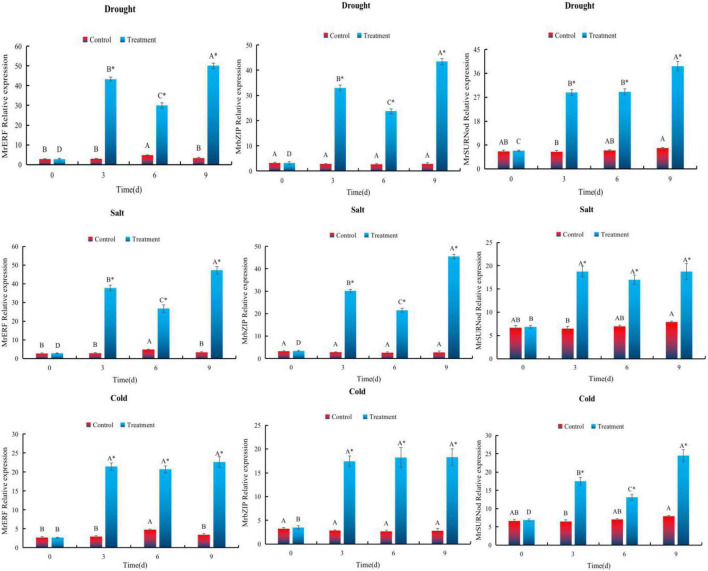
Expression patterns of *MrERF*, *MrbZIP*, and *MrSURNod* in leaf under abiotic stress. Different capital letters indicate significant differences under different treatments, and “*” indicates significant differences between control and treatment (*P* < 0.05).

To explore the expression of *MrERF*, *MrbZIP*, and *MrSURNod* in response to various stresses, we conducted qRT-PCR on seedlings of transgenic tobacco. The expression of *MrERF*, *MrbZIP*, and *MrSURNod* was induced strongly by water deficit, showing an overall upward trend with the duration of drought, and peaking (14. 77-, 15. 7-, and 4.9-fold higher than the non-stressed control, respectively) 9 days after the drought treatment started (Figure 5).

In the NaCl treatment, expression patterns of *MrERF* and *MrbZIP* were consistent with those during drought stress. The *MrERF* and *MrbZIP* expression reached the maximum (around 13.92- and 16.45-fold of the control, respectively) on 9th day. By contrast, *MrSURNod* expression was 2.90 times higher than that of the control, but there was no significant change at three time points during the stress (Figure 5). Therefore, NaCl can induce the rapid expression of *MrERF* or *MrbZIP* in leaves, but its effect on *MrSURNod* is relatively weaker than the other two.

For cold stress, the expression of the genes was significantly higher than that of the control. The expression of *MrERF* and *MrbZIP* did not change with the duration of the stress, but *MrSURNod* showed a gradual upward trend, reaching the maximum (around 3.09-fold of the control) after 9 days. Hence, low temperature can strongly induce the expression of *MrSURNod*, but its effect on *MrERF* and *MrbZIP* is relatively weak.

In summary, it is preliminarily concluded that *MrERF* and *MrbZIP* mainly respond to salt and drought, while *MrSURNod* mainly responds to drought and cold stress.

### Morphological and Physiological Changes in Transgenic Tobacco Under Abiotic Stress

#### Germination Rate of Transgenic Tobacco Seeds Under Abiotic Stress

**FIGURE 6:**
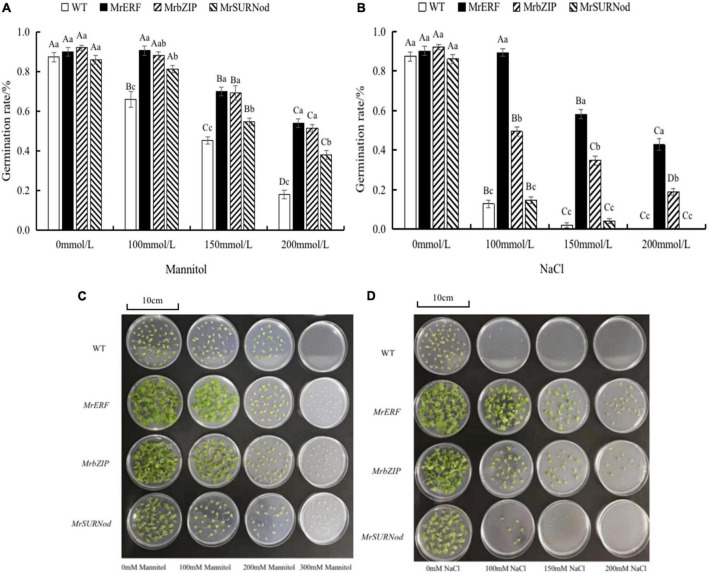
Germination of transgenic tobacco seeds treated with mannitol and NaCl. **(A,C)** Germination rate of transgenic tobacco seeds under mannitol treatment. **(B,D)** Germination rate of transgenic tobacco seeds under mannitol NaCl treatment. Different capital letters indicate significant differences under different treatments, and different lowercase letters indicate significant differences among different transgenic lines in the same treatment (*P* < 0.05).

The seed germination of transgenic tobacco lines was tested in this study (Figure 6). *MrERF*, *MrbZIP*, and *MrSURNod* are involved in the regulation of seed viability and can facilitate seed germination. Under normal conditions, the germination rate of WT or transgenic tobacco seeds was not significantly different. However, the leaf growth of three transgenic tobacco lines was better than that of WT, with *MrERF* having the best growth, followed by *MrbZIP*. The germination rate of the transgenic tobacco and WT seeds decreased with the increasing mannitol or NaCl concentration, but the germination rate of transgenic tobacco seeds was markedly higher than that of the WT seeds. The germination rate of transgenic tobacco lines with *MrERF* or *MrbZIP* was significantly higher than *MrSURNod*. Notably, transgenic tobacco with *MrSURNod* seeds did not germinate under 200 mM NaCl. Hence, *MrERF* and *MrbZIP* can improve the germination rate under osmotic stress or salt, whereas *MrSURNod* can improve seed germination rate under osmotic stress but only mild salt stress. In the cold treatment, WT and transgenic tobacco did not germinate.

#### Morphological Changes of Transgenic Tobacco Under Abiotic Stress

**FIGURE 7:**
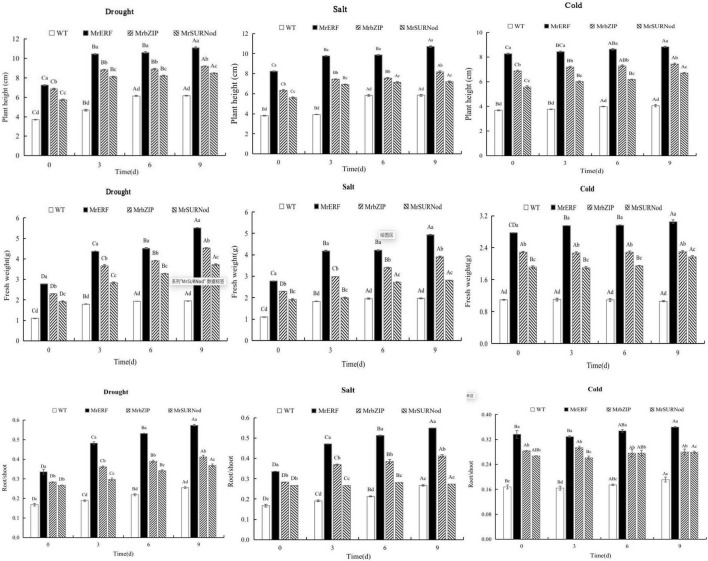
Plant height, biomass, and root/shoot of transgenic tobacco under abiotic stress. Different capital letters indicate significant differences under different treatments, and different lowercase letters indicate significant differences among different transgenic lines in the same treatment (*P* < 0.05).

As shown in Figure 7, the growth retardation (plant height, biomass, and root/shoot ratio) under various abiotic stresses was lesser in transgenic tobacco than in WT. Under drought, with the duration of the stress plant height, biomass, and root/shoot increased, with the maximum on day 9. Under NaCl treatment, plant height, biomass, and root/shoot ratio of transgenic tobacco with *MrERF* or *MrbZIP* increased significantly, no significant change was noted for *MrSURNod* transgenic tobacco after day 6. However, in the low-temperature treatment, the contrary result was observed, i.e., plant height, biomass, and root/shoot ratio of *MrSURNod* transgenic lines increased obviously with the stress duration, while transgenic tobacco with *MrERF* or *MrbZIP* showed no obvious change from day 6.

**FIGURE 8:**
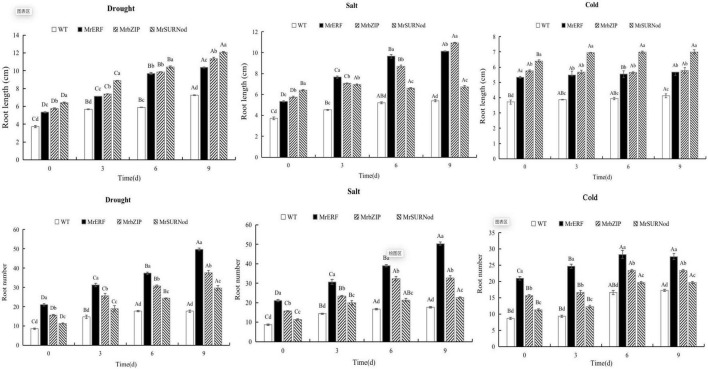
Effects of different stresses on root growth of tobacco. Different capital letters indicate significant differences under different treatments, and different lowercase letters indicate significant differences among different transgenic lines in the same treatment (*P* < 0.05).

We also noted that the root length and number were generally greater in transgenic plants than in WT (Figure 8). The transgenic plants with *MrSURNod* had the longest roots, followed by *MrbZIP*. With the duration of drought, the root length of *MrERF*, *MrbZIP*, and *MrSURNod* transgenic lines increased significantly, around 1. 48-, 1. 62-, and 1.73-fold of WT, respectively. Under salt stress, the root length of *MrERF* or *MrbZIP* transgenic lines increased, around 2.03- and 2.19-fold of WT, respectively, but the root length of *MrSURNod* transgenic plants increased only slightly. The root number increased gradually under drought or salt stress. The root number of transgenic tobacco with *MrERF* was the largest, around 2.76-fold of WT, followed by *MrbZIP* (2.09-fold increase), and *MrSURNod* was the smallest. In the cold treatment, the root length of *MrSURNod* transgenic tobacco increased significantly, but the other lines showed no significant change.

#### Physiological Changes in Transgenic Tobacco Under Abiotic Stress

**FIGURE 9:**
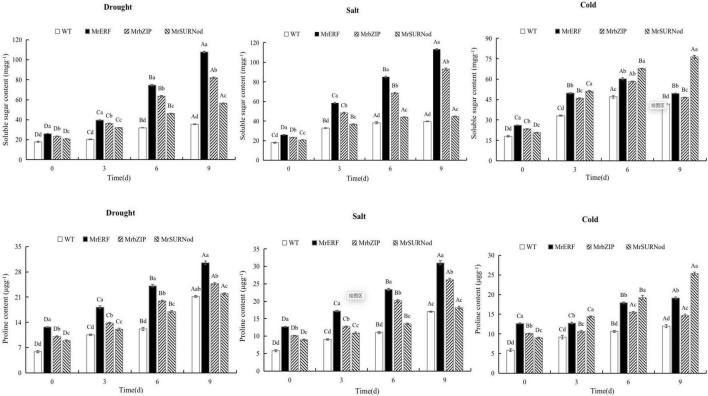
Effects of different stresses on osmotic adjustment substance of tobacco. Different capital letters indicate significant differences under different treatments, and different lowercase letters indicate significant differences among different transgenic lines in the same treatment (*P* < 0.05).

**FIGURE 10:**
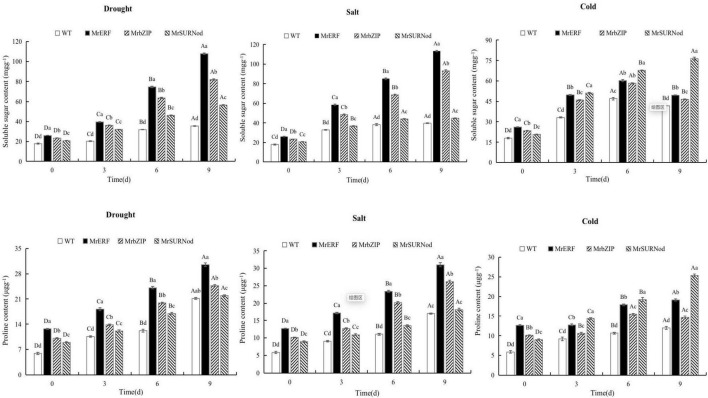
Effects of different stresses on antioxidant enzyme activity of tobacco. Different capital letters indicate significant differences under different treatments, and different lowercase letters indicate significant differences among different transgenic lines in the same treatment (*P* < 0.05).

**FIGURE 11:**
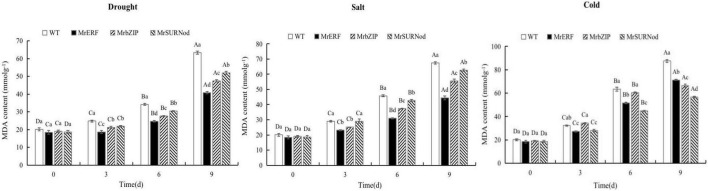
Effects of different stresses on MDA content of tobacco. Different capital letters indicate significant differences under different treatments, and different lowercase letters indicate significant differences among different transgenic lines in the same treatment (*P* < 0.05).

In addition to morphological changes, we also quantified the stress-related physiological and biochemical indicators, including osmoregulation substances (Figure 9), protective enzymes (Figure 10), and MDA (Figure 11). Significant increases in soluble sugars, proline content, SOD, and POD were observed under abiotic stress. With the prolonged drought and salt stress, the osmolyte content and antioxidant enzyme activities showed an upward trend and peaked after 9 days of stress. This finding indicates that the introduction of exogenous transcription factors can increase the accumulation of soluble sugars and proline and activity of the antioxidant enzyme in transgenic lines, thereby improving the resistance to drought and salt stress. These indices were highest in transgenic tobacco with *MrERF*, followed by *MrbZIP* and *MrSURNod.* However, with the duration of salt stress, the soluble sugar and proline contents and activities of SOD and POD in *MrERF* or *MrbZIP* transgenic tobacco leaves increased significantly, whereas in *MrSURNod* transgenic tobacco leaves, there was no significant increase after 6 days of stress. In the cold treatment, these indices first increased (peaking at day 6) and then decreased in *MrERF* or *MrbZIP* transgenic tobacco, but continued to increase significantly in *MrSURNod* transgenic tobacco. Overall, these results indicate that the salt tolerance of transgenic tobacco with *MrERF* or *MrbZIP* is stronger than that of *MrSURNod*, whereas the cold tolerance of the transgenic line with *MrSURNod* is the highest of the three lines. This was further verified by MDA content.

Under abiotic stress, the MDA content in WT exhibited a significant rise when compared with three transgenic tobacco lines, and reached the maximum after 9 days of stress, indicating that the severity of damage was lower in transgenic plants than in WT. Accumulation of MDA in *MrERF* transgenic plants was the lowest, followed by *MrbZIP*. Its accumulation in *MrERF* or *MrbZIP* transgenic lines under salt was relatively low, and the accumulation in *MrSURNod* was relatively high, indicating that the cell membrane damage in *MrSURNod* transgenic plants was more severe. However, the opposite result was found in the cold treatment, with a degree of damage lower in *MrSURNod* transgenic plants than *MrERF* or *MrbZIP* transgenic plants.

Based on the results of morphological and physiological parameters, transgenic tobacco performed better than WT in all aspects, with *MrERF* transgenic plants performing best, followed by *MrbZIP*. Overall, it is speculated that transgenic plants with *MrERF* or *MrbZIP* have stronger drought and salt tolerance than *MrSURNod*, whereas transgenic tobacco with *MrSURNod* is more cold tolerant.

## Discussion

It has been well documented that they are involved not only in plant growth and development by regulating plant hormones (e.g., ABA) but also involved in the response to abiotic stresses, e.g., drought, low temperature, and salinity. Compared with other cultivated medics (*M. sativa*, *M. truncatula*, etc.), the AP2/ERF and bZIP family genes in *M. ruthenica* were more abundant (Wang et al., 2021). In addition, the SURNod19 gene family is annotated in NCBI as up-regulated by stress. Although SURNod19 has been identified as a cold-induced gene by the gene-chip analysis (Nakamura et al., 2013), it has not been reported whether it responds to other abiotic stresses. Here, we have selected a gene from each of the three gene families mentioned above. Our study highlights the importance of *MrERF*, *MrbZIP*, and *MrSURNod* in plant growth and the responses to abiotic stresses.

In our study, the three genes (*MrERF*, *MrbZIP*, and *MrSURNod*) with homology to ERF, bZIP, and SURNod19, respectively, were among the DEGs in response to drought stress in *M. ruthenica*. The deduced proteins (*MrERF* and *MrbZIP*) showed high similarity with, respectively, *AtERF034* and *AtbZIP46* from *A. thaliana*. Subcellular localization analysis implied the possible localization of *MrERF* and *MrbZIP* in the nucleus and *MrSURNod* in the chloroplast. Up-regulation of these genes improved the drought tolerance of *M. ruthenica*. The expression patterns of the three genes in transgenic tobacco lines were consistent in showing an upward trend under drought, cold, and NaCl stress. This is similar to the expression patterns of other transcription factors related to plant stress resistance, such as *OsERF65* (Zhao et al., 2021) and *VrbZIP52* (Wang L. et al., 2018), indicating that the genes may be related to plant stress responses. Salt and drought stress significantly induced the expression of *MrERF* and *MrbZIP* in tobacco, suggesting an important role of these two genes in plant responses to salt and drought stresses. The *MrSURNod* was induced by drought or cold stress, suggesting a regulatory function in response to drought and low temperature.

### *MrERF*, *MrbZIP*, and *MrSURNod* Are Involved in Plant Growth and Development

The ERF transcription factors respond to the ethylene (ETH) signal and play an important role in ethylene-dependent physiological activities (Bolt et al., 2017). For example, the tomato *SlERF52* affects the abscission of pedicel (Nakano et al., 2014), overexpression of *AtERF019* can slow down plant growth and delay senescence (Scarpeci et al., 2017), *TINY* causes growth retardation (Xie et al., 2019), and the rice *OsEATB* can inhibit internode elongation and thereby result in dwarfed phenotype (Qi et al., 2011). The bZIP transcription factors also regulate growth, senescence, flower development, and seed maturation, e.g., *AtbZIP9* and *AtbZIP46* are involved in regulating leaf development. However, there are few studies on the effect of the *SURNod19* family genes on growth and development. In our study, *MrERF*, *MrbZIP*, and *MrSURNod* belong to the same families as the example genes listed above, but they have distinct functions. Under non-stress conditions, transgenic tobacco over-expressing one of the three genes showed different growth and development characteristics than WT. For example, the plant height, biomass, and seed yield of tobacco with *MrERF*, *MrbZIP*, or *MrSURNod* were higher than those of WT, and all the three transgenic lines flowered earlier and had a shorter growth period than WT, which is speculated to be one of the ways to cope with stress. These findings were similar to *PpcERF5*, whose overexpression was associated with earlier flowering in *Arabidopsis* (Gao et al., 2021). The three genes also promoted branching in transgenic tobacco. In addition, *MrERF*, *MrbZIP*, and *MrSURNod* were beneficial to root development. Both *MrERF* and *MrbZIP* increased root length and number, whereas *MrSURNod* mainly promoted root elongation. Enhanced root growth and development is conducive to increasing the shoot height and biomass. In summary, the three genes tested are involved in plant growth and development, and their regulatory mechanisms need to be explored further.

### *MrERF*, *MrbZIP*, and *MrSURNod* Improved Abiotic Stress Resistance by Changing Plant Morphology

In addition to promoting the growth and development of plants, the *MrERF*, *MrbZIP*, and *MrSURNod* may also serve as regulators of abiotic stress responses by altering gene expression. Water and nutrient absorption is strongly correlated with root morphology and distribution in soil. Deep roots distributed widely and with more branches are beneficial for water absorption and utilization, i.e., the water absorption is increased by increasing the root contact area with soil particles (Jones and Dolan, 2012). It has been shown that root distribution down the soil profile is an important determinant of drought adaptability (Grieder et al., 2014). Moreover, the increase in root number also can improve the drought resistance of plants (Henry et al., 2011). In our study, the root length as well as number were significantly higher in transgenic plants (*MrERF*, *MrbZIP*, and *MrSURNod*) than in WT, indicating that *MrERF*, *MrbZIP*, and *MrSURNod* can improve the plant capacity to utilize soil moisture by increasing the number and length of roots in horizontal and vertical directions. However, the roots of transgenic tobacco over-expressing *MrSURNod* were longer in length but fewer in number, whereas transgenics over-expressing the other two genes showed the reverse results. Transgenic tobacco over-expressing *MrERF* had the largest number of roots, and its shoot height and biomass and root/shoot ratio were significantly higher than those of wild type and other transgenic tobacco lines. In summary, *MrERF* and *MrbZIP* promote root elongation and branching (root number), whereas *MrSURNod* mainly promotes root elongation, but all these responses are likely to enhance adaptation to abiotic stresses.

The root/shoot ratio reflects the environment adaptability of plants. Increased root/shoot ratio was associated with dehydration (Du et al., 2020). Similarly, in our study, the root/shoot ratio of tobacco lines over-expressing *MrERF*, *MrbZIP*, or *MrSURNod* was significantly higher than that of WT. This resource allocation in plants is a strategy associated with enhanced adaptation to drought.

Additionally, we found the three genes to be beneficial to seed germination under stress, except in cold environments. The over-expression of *MrERF* was linked to the highest germination rate, and its function appeared to be similar to *PsnERF75* (Wang S. J. et al., 2018) and *BoERF1* (Jiang et al., 2018).

### *MrERF*, *MrbZIP*, and *MrSURNod* Improved Osmotic Adjustment and Antioxidant Capacity to Enhance Resistance to Abiotic Stresses

Previous studies have shown significantly higher root length and number in the three over-expression lines compared with WT under stress, indicating that *MrERF*, *MrbZIP*, and *MrSURNod* play important roles in resistance to abiotic stress. The physiological parameters of transgenic lines under abiotic stress confirmed that *MrERF*, *MrbZIP*, and *MrSURNod* play an important positive regulatory role in plant abiotic stress response. Regardless of the growth conditions, the content of osmoregulation substances and the activity of antioxidant enzymes were higher in transgenic plants than in WT, implying that the transgenic lines may have more efficient osmoregulation and antioxidant systems. MDA is an important product of lipid peroxidation in the cell membranes, and its concentration varies in response to biotic and abiotic stresses. Here, we found that less MDA was generated in transgenic than WT tobacco seedlings, suggesting that over-expression of the tested genes can significantly improve plant tolerance to stress.

The osmolyte content and antioxidant enzyme activity in tobacco lines over-expressing *MrERF* or *MrbZIP* were higher than in the line over-expressing *MrSURNod*. The stress damage in *MrERF* or *MrbZIP* transgenic plants under drought and salt stress was light, indicating strong resistance, which is similar to chrysanthemum *CmERF053* (Nie et al., 2018), *OsERF71* (Ahn et al., 2017), sweet potato *IbRAP2-12* (Li et al., 2019), *OsbZIP71* (Liu et al., 2018), *StbZIP65* (Zhao et al., 2020), *AtbZIP36*, *AtbZIP37*, and *AtbZIP38* (Takuya et al., 2010). *MrERF* performed better in drought and salt tolerance than *MrbZIP*. The SURNod 19 family has been identified in *Brachypodium distachyon* (Martin et al., 2018), but its role in the plant response to the stress is unclear, although it has been considered a cold-inducible gene in *Bromus inermis* Leyss (Nakamura et al., 2013). In our study, the expression of this gene responded mainly to drought and to a small extent to cold stress. Fewer roots in tobacco over-expressing this gene might have been the reason for their weak salt tolerance. Overall, the three genes from *M. ruthenica* can enhance the resistance of transgenic tobacco to stress, with *MrERF* and *MrbZIP* being most effective in improving the resistance of tobacco to stress.

## Conclusion

Through comprehensive bioinformatics, morphological, physiological, and transcriptomic analyses of the transgenic tobacco plant under various abiotic stresses, we have revealed the critical role of *MrERF, MrbZIP*, and *MrSURNod* in plant growth and abiotic stress responses. In addition to plant height and biomass, the over-expression of *MrERF*, *MrbZIP*, and *MrSURNod* promoted the production of branches and improved the yield of tobacco seeds. Furthermore, these genes were associated with early flowering and shortened growth periods in tobacco, which is one of the ways for transgenic tobacco to cope with stress. *MrERF*, *MrbZIP*, and *MrSURNod* also enhanced root development, which is conducive to resisting stress. Also, under stress, different transgenic lines enhanced stress resistance by increasing the osmolyte content and antioxidant enzyme activity.

In summary, *MrERF*, *MrbZIP*, and *MrSURNod* positively modulate stress resistance. *MrERF* and *MrbZIP* can improve drought and salt tolerance of plants, whereas *MrSURNod* is beneficial to improving drought and cold resistance. Transgenic tobacco over-expressing *MrERF* or *MrbZIP* showed better resistance than transgenic tobacco over-expressing *MrSURNod.*

## Data Availability Statement

The datasets presented in this study can be found in online repositories. The names of the repository/repositories and accession number(s) can be found in the article/Supplementary Material.

## Author Contributions

FLS and RNW conceived the original screening and research plans. FLS supervised the experiments. RNW and BX designed and performed the experiments. BX analyzed the data. RNW conceived the project and wrote the article with the contributions of all the authors. RNW agreed to serve as the author responsible for contact and ensures communication. All authors reviewed and approved the final manuscript.

## Conflict of Interest

The authors declare that the research was conducted in the absence of any commercial or financial relationships that could be construed as a potential conflict of interest.

## Publisher’s Note

All claims expressed in this article are solely those of the authors and do not necessarily represent those of their affiliated organizations, or those of the publisher, the editors and the reviewers. Any product that may be evaluated in this article, or claim that may be made by its manufacturer, is not guaranteed or endorsed by the publisher.

## Footnotes

1
http://web.expasy.org

2
http://wolfpsort.org/

3
http://www.cbs.dtu.dk/services/TMHMM/

4
http://smart.embl-heidelberg.de/
