# Supplementary material for: MrERF, MrbZIP, and MrSURNod of Medicago ruthenica Are Involved in Plant Growth and Abiotic Stress Response
Source: Front Plant Sci. 2022 Jun 2;13:907674. doi: 10.3389/fpls.2022.907674 (PMC9203031; doi:10.3389/fpls.2022.907674)
Supplement: Supplementary file 1 [file Image_1.pdf]

A

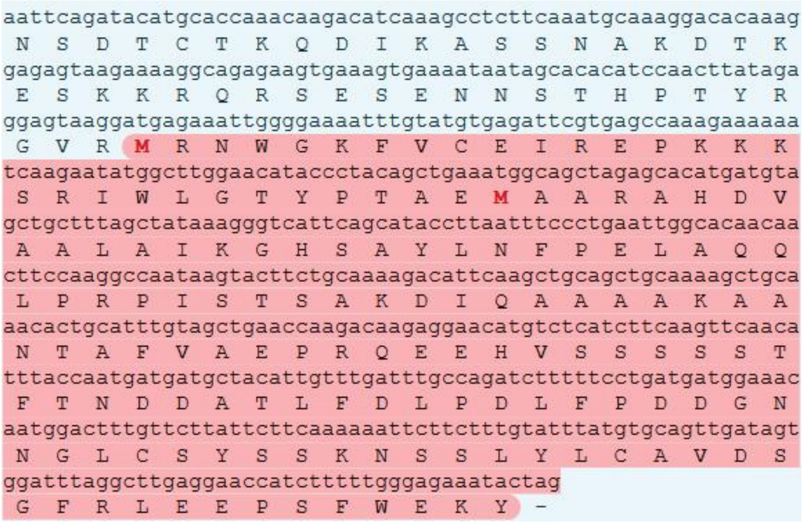

B

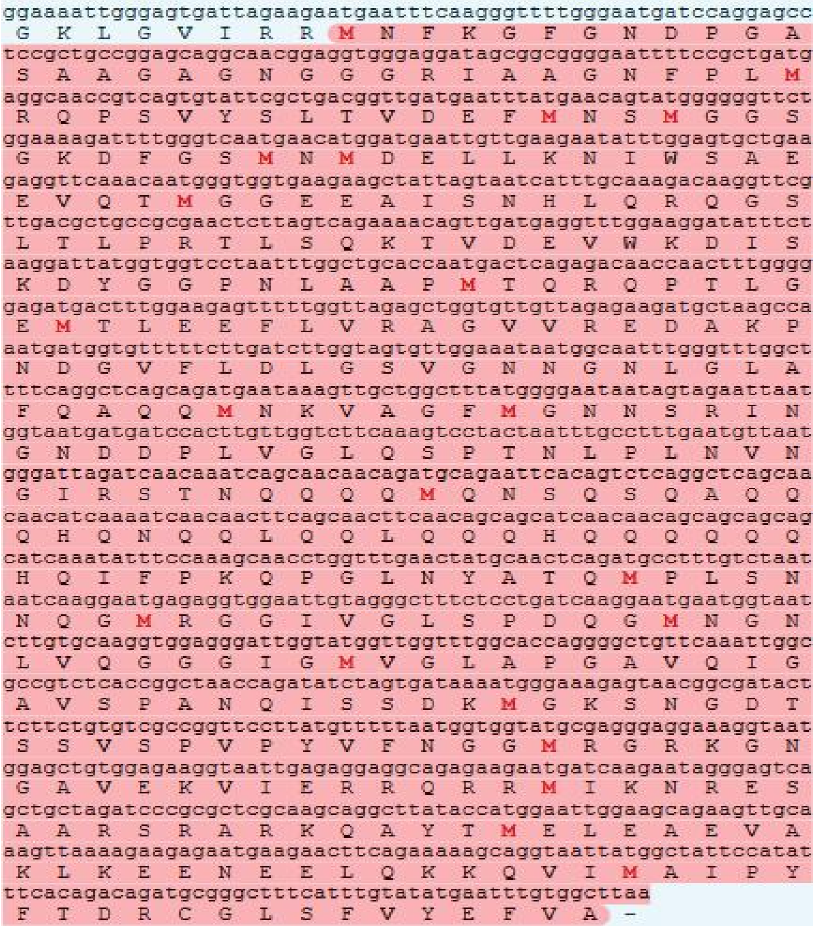

C

```
cagatcaacatgagttttgtatcaagagatttggtattatcattgccaatatttctgctg
 Q  I  N  M  S  F  V  S  R  D  L  V  L  S  L  P  I  F  L  L
ttgttaggcatacaatgctcaggttcttttgtgaagactgaagctaagataaaatcagct
 L  L  G  I  Q  C  S  G  S  F  V  K  T  E  A  K  I  K  S  A
gtatttttgtcccccaaatttgagctaggacctggatcagttgtcaatggatattattat
 V  F  L  S  P  K  F  E  L  G  P  G  S  V  V  N  G  Y  Y  Y
gatattggctttccaagagggcatgtagcactaaagagtttcaatgctgaagtagttgac
 D  I  G  F  P  R  G  H  V  A  L  K  S  F  N  A  E  V  V  D
gaatcagggaaacctgtacctctccatgaaacttatctacatcattggattgttgtaaga
 E  S  G  K  P  V  P  L  H  E  T  Y  L  H  H  W  I  V  V  R
tatcatcaaagtaaacatgtgacacaaaaaagacatgttggatacaaaatgcttcaaaac
 Y  H  Q  S  K  H  V  T  Q  K  R  H  V  G  Y  K  M  L  Q  N
tctgataatgttcttattaggaacagtggcatgtgtcaaggaaatactcttggacagtac
 S  D  N  V  L  I  R  N  S  G  M  C  Q  G  N  T  L  G  Q  Y
tttggccttggatccgaaacacgtggaacagcgaccgatattccggaccctttcggtata
 F  G  L  G  S  E  T  R  G  T  A  T  D  I  P  D  P  F  G  I
gagataggtaatcctgtagagattccagaaggttttgaggagaaatggttgcttaatgtt
 E  I  G  N  P  V  E  I  P  E  G  F  E  E  K  W  L  L  N  V
catgccattgatacgcgcggcacagaggataaattagggtgcactgagtgtaagtgtgac
 H  A  I  D  T  R  G  T  E  D  K  L  G  C  T  E  C  K  C  D
ctttataatgttacagtggatgaatatggaaggtctattaggccagattataaaggaggt
 L  Y  N  V  T  V  D  E  Y  G  R  S  I  R  P  D  Y  K  G  G
ttgttatgttgttatgattctaaacagtgcaagttgaaggaaggttttgagggtccaaaa
 L  L  C  C  Y  D  S  K  Q  C  K  L  K  E  G  F  E  G  P  K
agaagcctatacttaagatacacggttaaatgggttgattgggatgaatttatagtgcct
 R  S  L  Y  L  R  Y  T  V  K  W  V  D  W  D  E  F  I  V  P
gttaagatatatatcattgatgttactgatactttgaaactatcagataattcaaaagga
 V  K  I  Y  I  I  D  V  T  D  T  L  K  L  S  D  N  S  K  G
atgaactcagatcatgattgcaagaatgagtatcaagttgaatcttgcagcagagaccat
 M  N  S  D  H  D  C  K  N  E  Y  Q  V  E  S  C  S  R  D  H
aaggagggaaattcctgtgttcatgtgaagaggacaagtctcccatttcaaactggtggc
 K  E  G  N  S  C  V  H  V  K  R  T  S  L  P  F  Q  T  G  G
tatgtgatctatgctgtagctcatcaacattcaggtggaattggatcaactctatatgga
 Y  V  I  Y  A  V  A  H  Q  H  S  G  G  I  G  S  T  L  Y  G
caggatggg
 Q  D  G
```

Supplementary Figure 1 Amino sequence (A: *MrERF*, B: *MrbZIP*, C; *MrSURNod*)

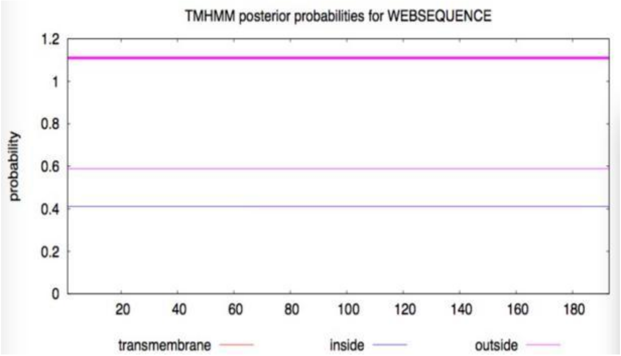

MrERF

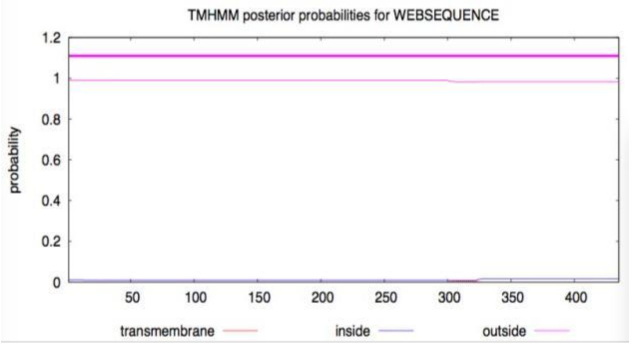

MrbZIP

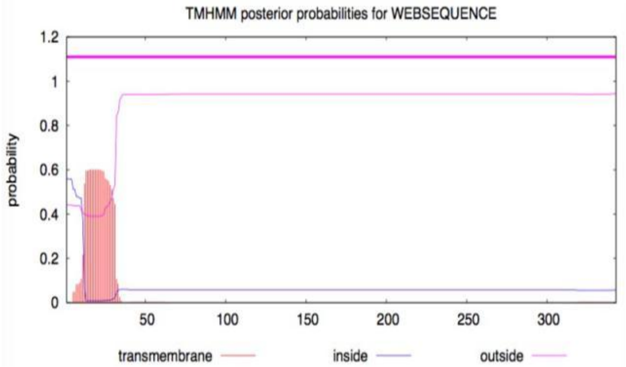

MrSURNod

Supplementary Figure S2 Transmembrane region prediction of protein(*MrERF*, *MrbZIP*, *MrSURNod*)

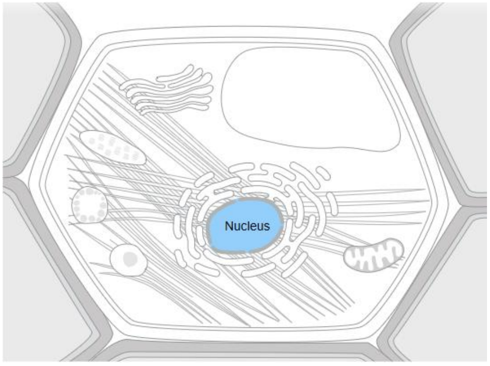

MrERF, MrbZIP

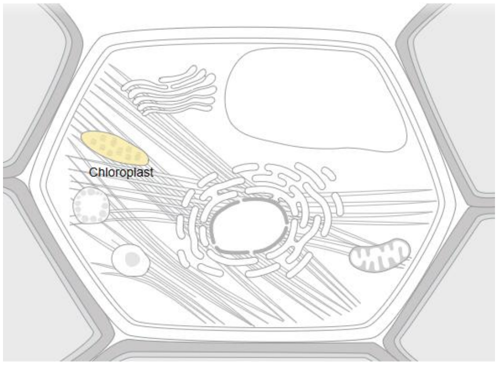

MrSURNod

Supplementary Figure S3 Predicted localization of protein(*MrERF*, *MrbZIP*, *MrSURNod*)

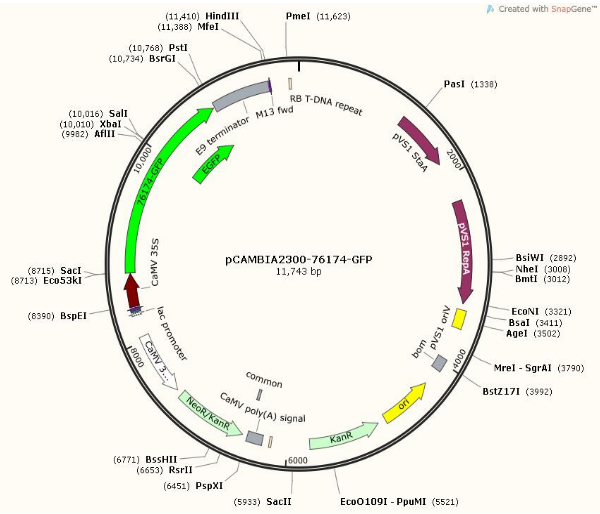

MrbZIP

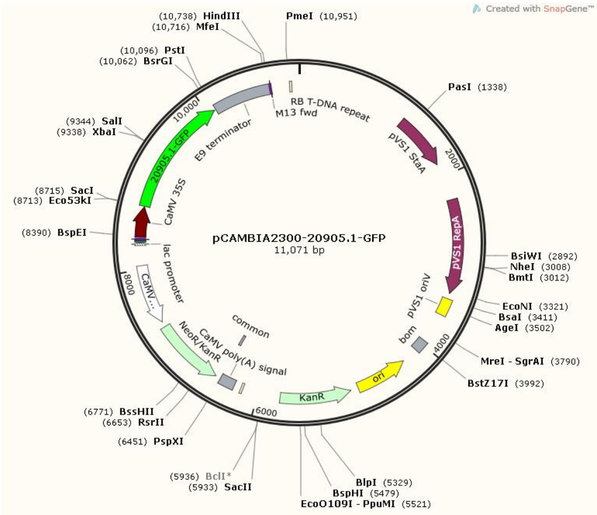

MrERF

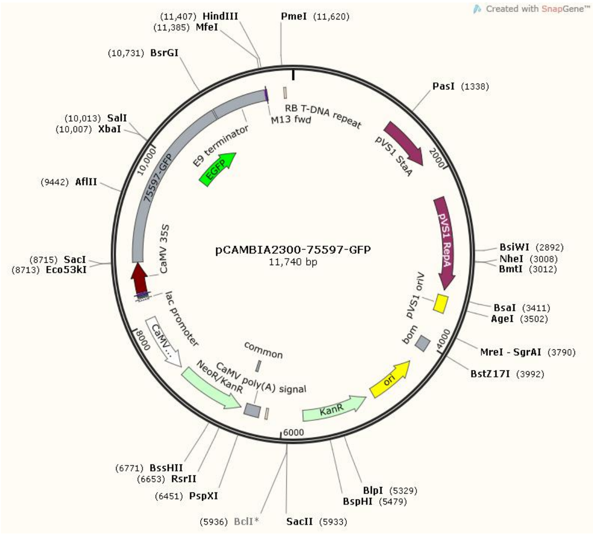

MrSURNod

Supplementary Figure S4　Construction of target genes expression vectors

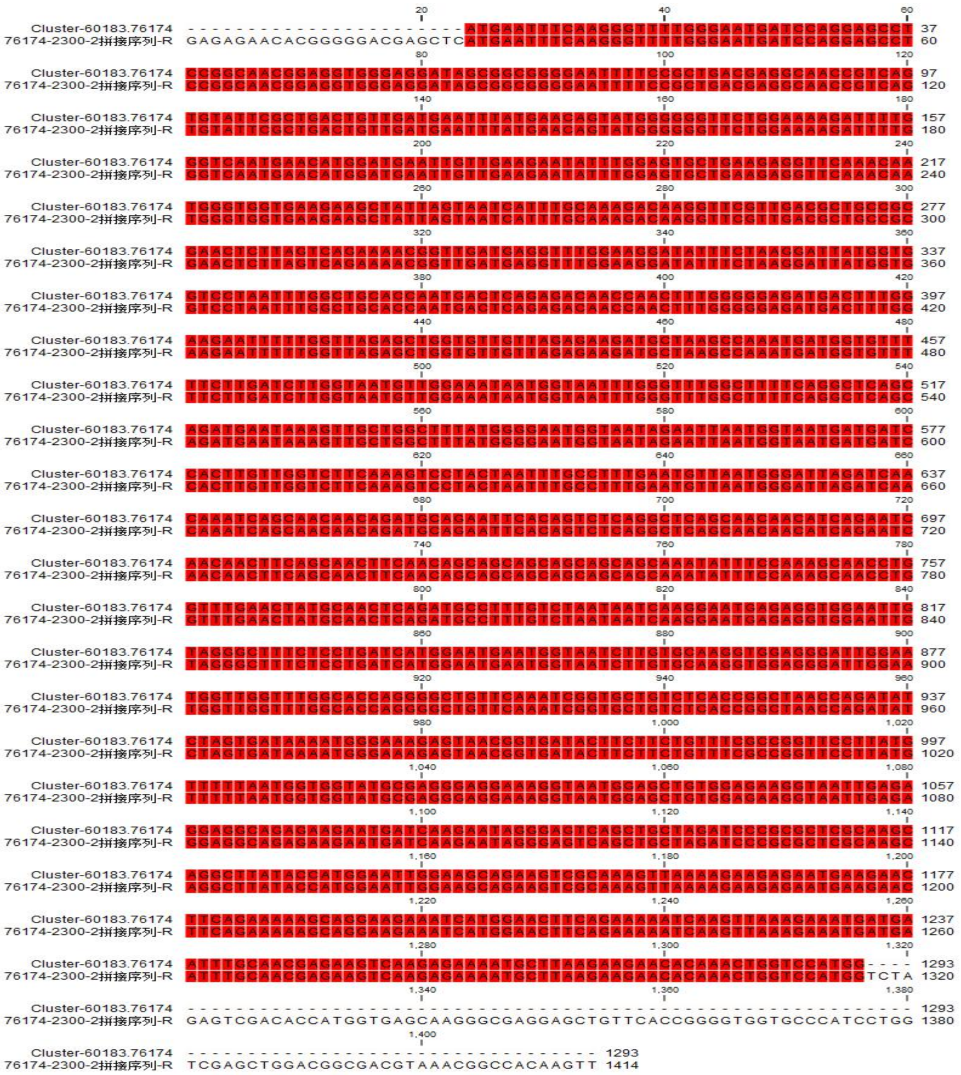

pCAMBIA2300-*MrbZIP*-GFP

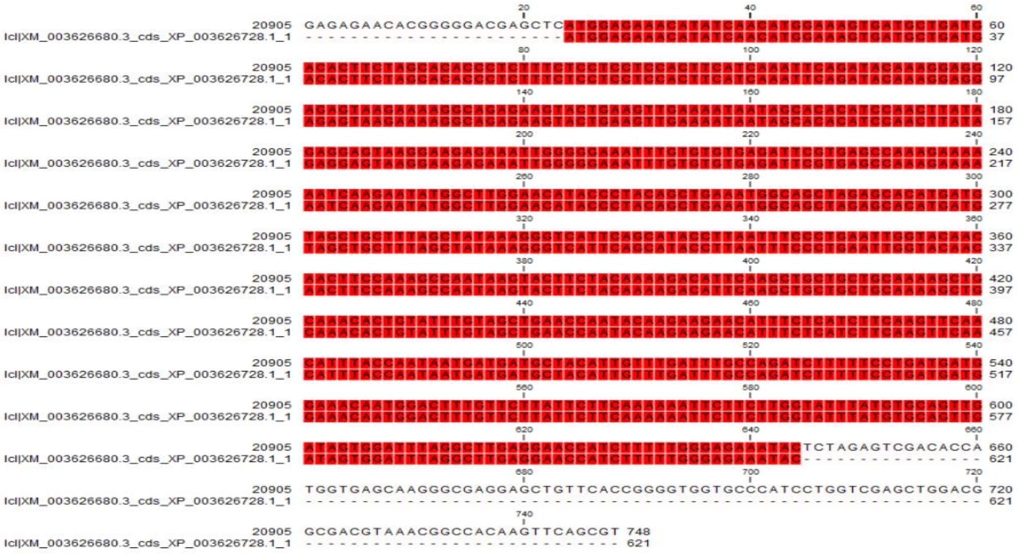

pCAMBIA2300-*MrERF*-GFP

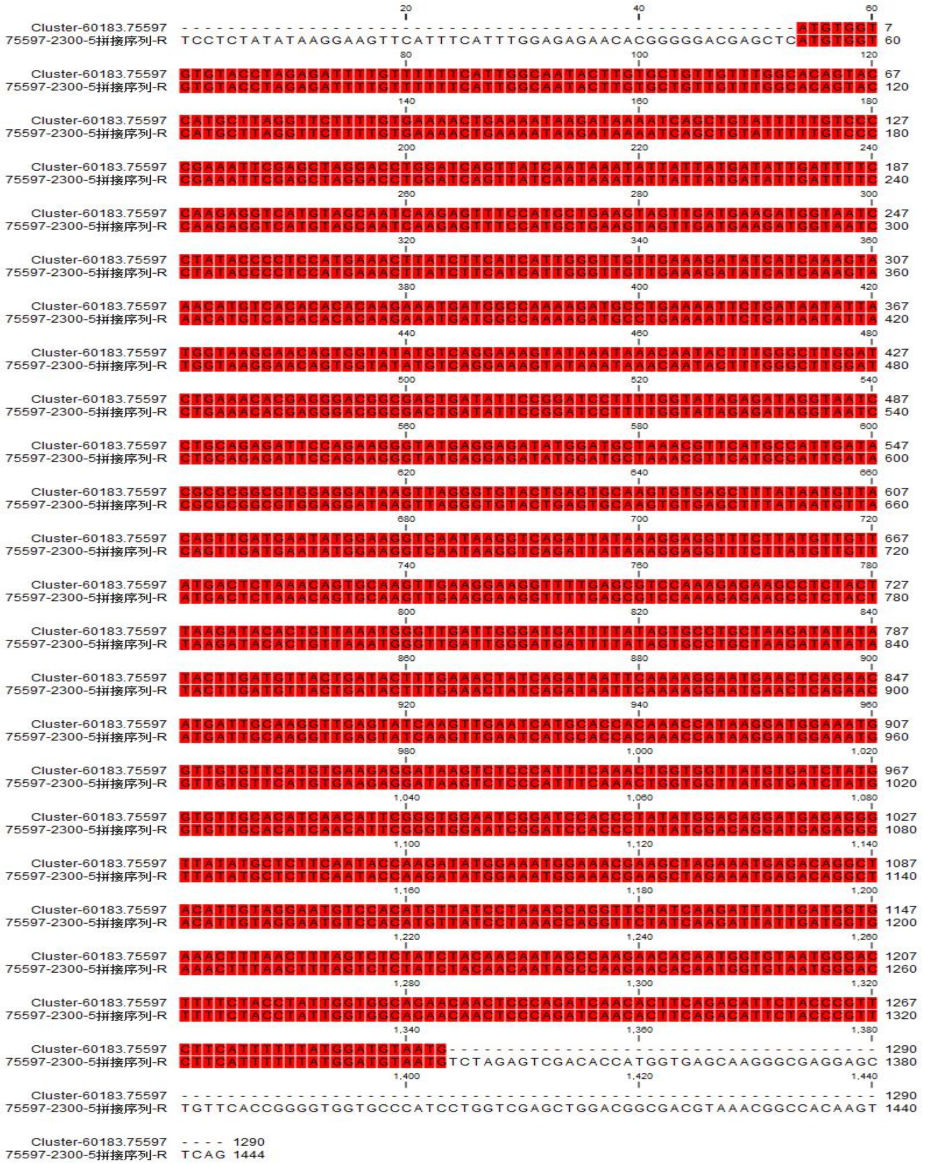

pCAMBIA2300-*MrSURNod*-GFP

Supplementary Figure S5 Sequencing results of vector

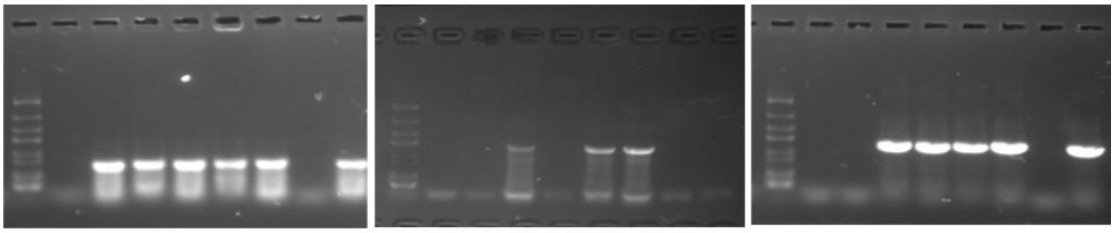

M CK,1, 2, 3, 4, 5, 6, 7 M CK,1, 2, 3, 4, 5, 6, 7 M CK, 1, 2, 3, 4, 5, 6, 7

MrbZIP-2300 MrERF-2300 MrSURNod-2300

Supplementary Figure.S6 Bacterial test chart

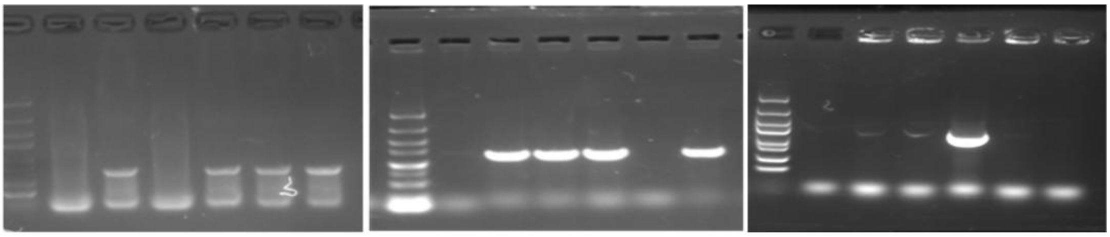

M CK, 1, 2, 3, 4, 5 M CK, 1, 2, 3, 4, 5 M CK, 1, 2, 3, 4, 5

MrbZIP MrERF MrSURNod

Supplementary Figure S7 Detection of Agrobacterium
