# Supplementary figures and images for: MrERF, MrbZIP, and MrSURNod of Medicago ruthenica Are Involved in Plant Growth and Abiotic Stress Response
Source: Front Plant Sci. 2022 Jun 2;13:907674. doi: 10.3389/fpls.2022.907674 (PMC9203031; doi:10.3389/fpls.2022.907674)

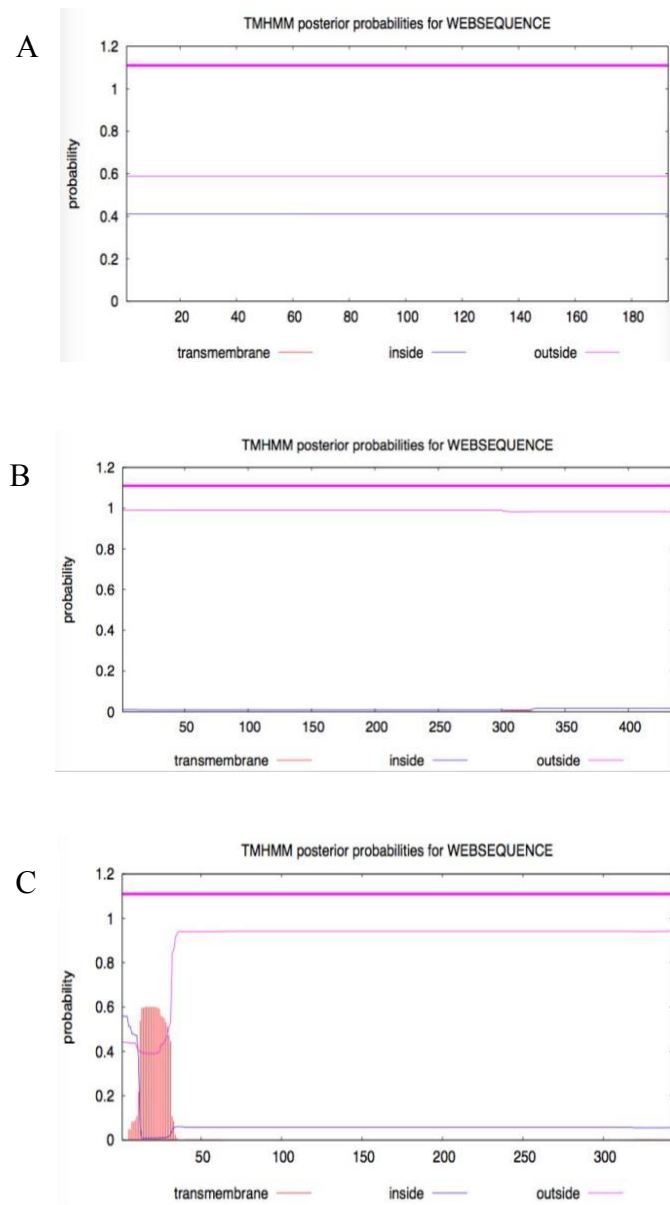

Supplementary Figure 2 Transmembrane region prediction of protein (A: *MrERF*, B: *MrbZIP*, C: *MrSURNod*)

Supplement: Supplementary file 2 [file Image_2.pdf]

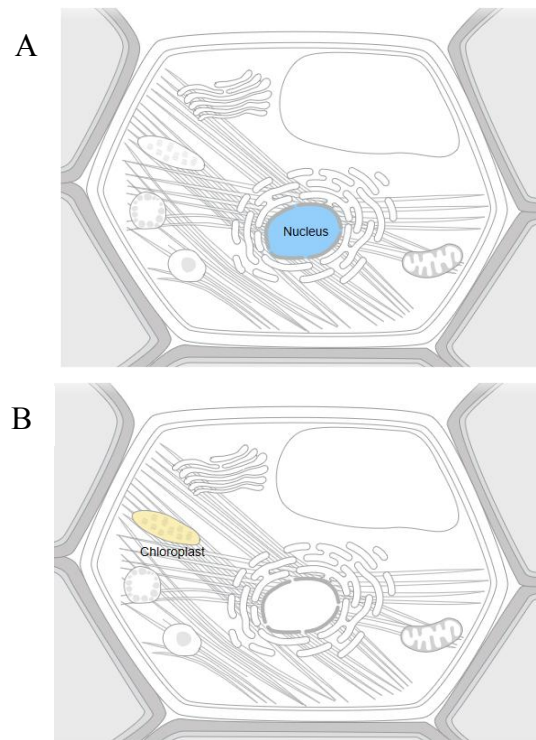

Supplementary Figure 3 Predicted localization of protein (A: *MrERF* and *MrbZIP*, B: *MrSURNod*)

Supplement: Supplementary file 3 [file Image_3.pdf]

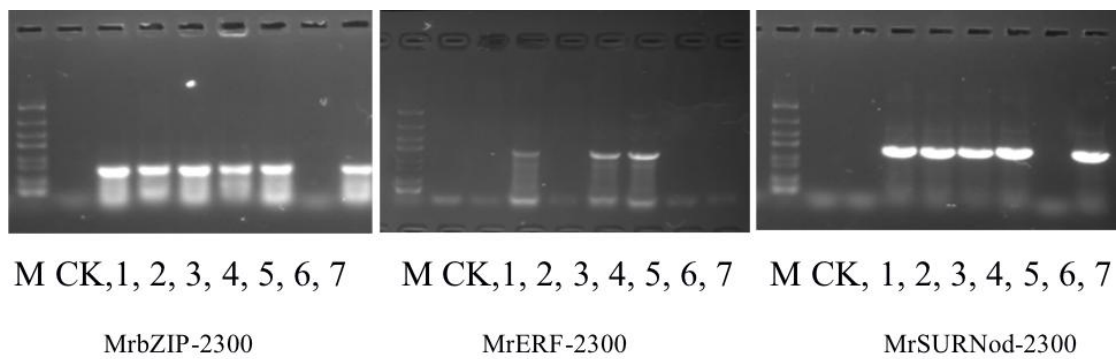

Supplementary Figure.S6 Bacterial test chart

Supplement: Supplementary file 6 [file Image_6.pdf]

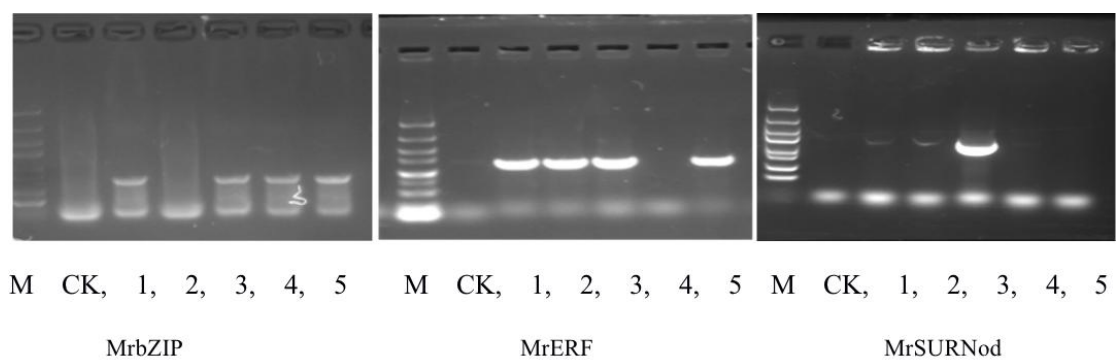

Supplementary Figure S7 Detection of Agrobacterium

Supplement: Supplementary file 7 [file Image_7.pdf]
