# Supplementary material for: MrERF, MrbZIP, and MrSURNod of Medicago ruthenica Are Involved in Plant Growth and Abiotic Stress Response
Source: Front Plant Sci. 2022 Jun 2;13:907674. doi: 10.3389/fpls.2022.907674 (PMC9203031; doi:10.3389/fpls.2022.907674)
Supplement: Supplementary file 4 [file Image_4.pdf]

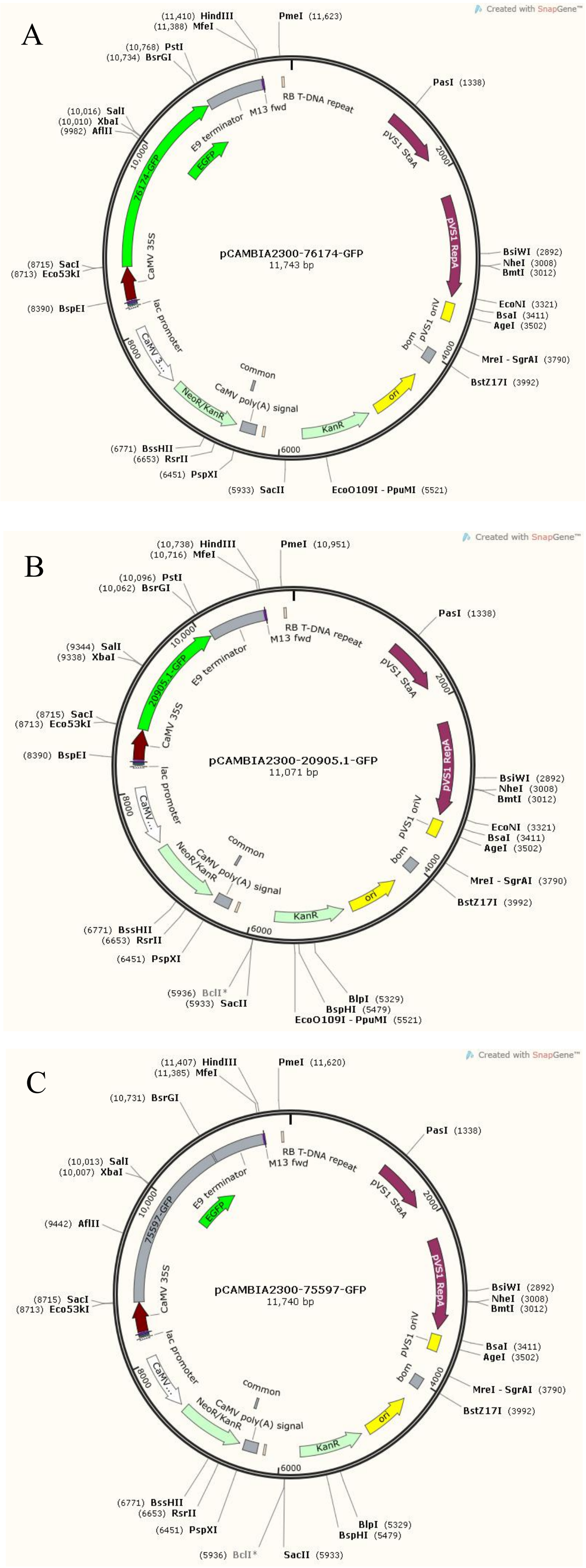

Supplementary Figure 4 Construction of target genes expression vectors (A (pCAMBIA2300-76174-GFP): *MrbZIP* expression vector; B (pCAMBIA2300-20905.1-GFP): *MrERF* expression vector; C (pCAMBIA2300-75597-GFP): *MrSURNod* expression vector)
