# Supplementary material for: MrERF, MrbZIP, and MrSURNod of Medicago ruthenica Are Involved in Plant Growth and Abiotic Stress Response
Source: Front Plant Sci. 2022 Jun 2;13:907674. doi: 10.3389/fpls.2022.907674 (PMC9203031; doi:10.3389/fpls.2022.907674)
Supplement: Supplementary file 5 [file Image_5.pdf]

A

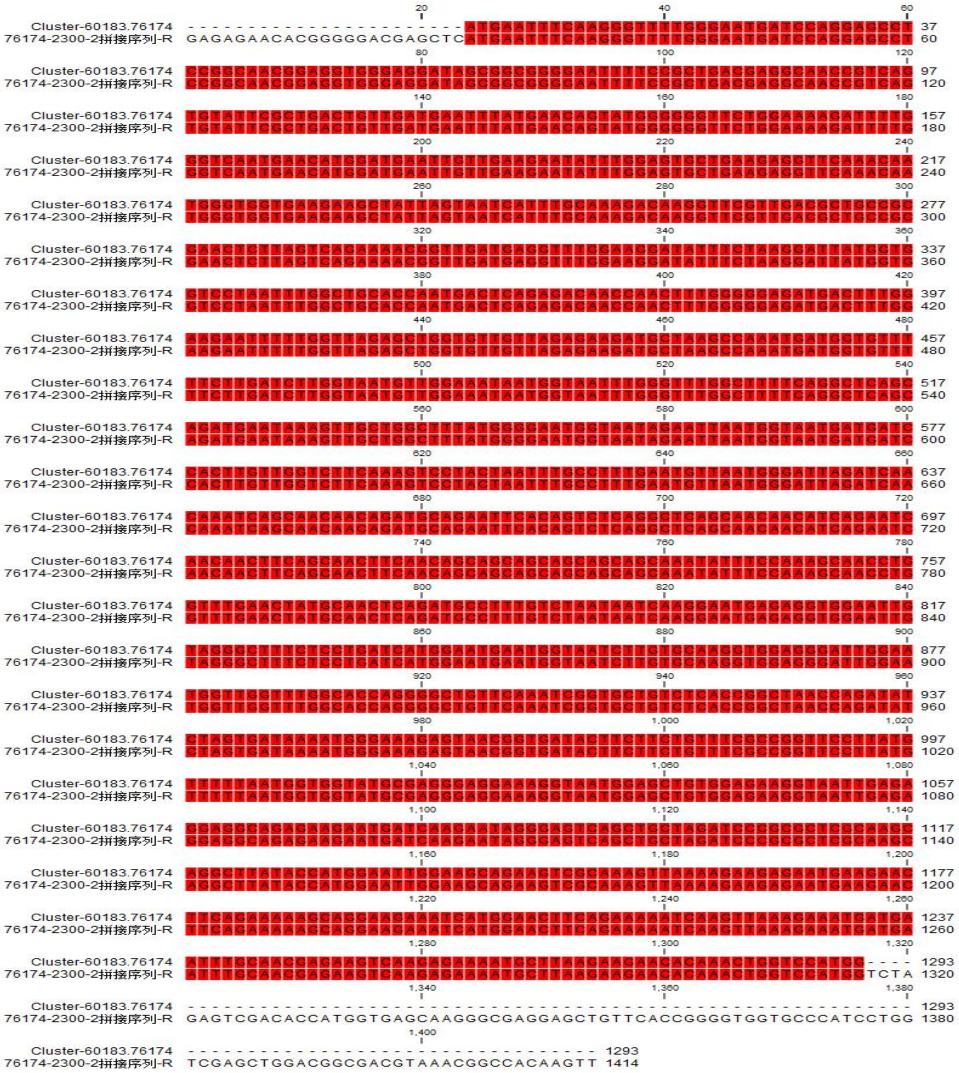
Cluster-60183.76174
76174-2300-2拼接序列-R
GAGAGAACACGGGGGACGAGCTC
TCTA
GAGTCGACACCATGGTGAGCAAGGGCGAGGAGCTGTTCACCGGGGTGGTGCCCATCCTGG
TCGAGCTGGACGGCGACGTAAACGGCCACAAGTT

B

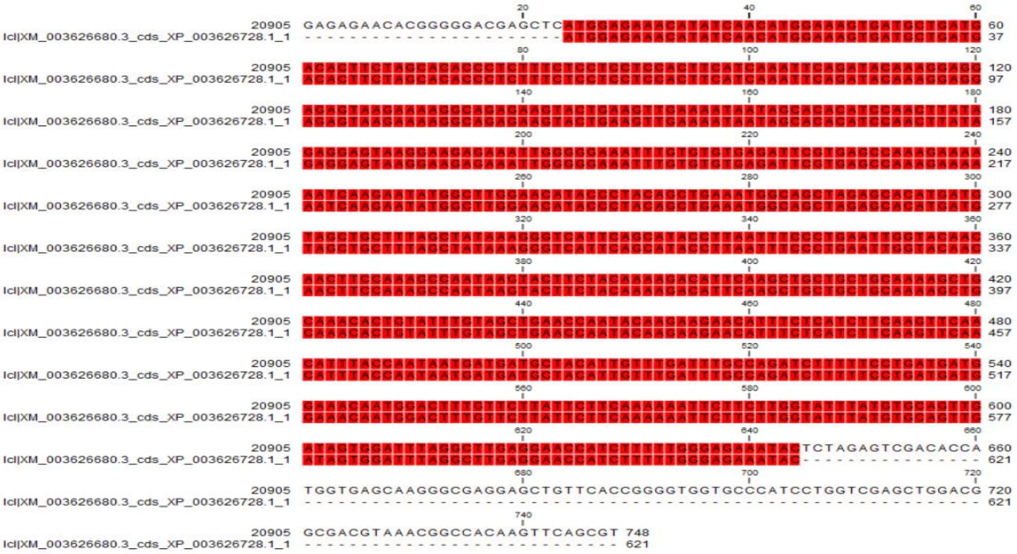
20905
lcl|XM_003626680.3_cds_XP_003626728.1_1
GAGAGAACACGGGGGACGAGCTC
TCTAGAGTCGACACCA
TGGTGAGCAAGGGCGAGGAGCTGTTCACCGGGGTGGTGCCCATCCTGGTCGAGCTGGACG
GCGACGTAAACGGCCACAAGTTCAGCGT

C

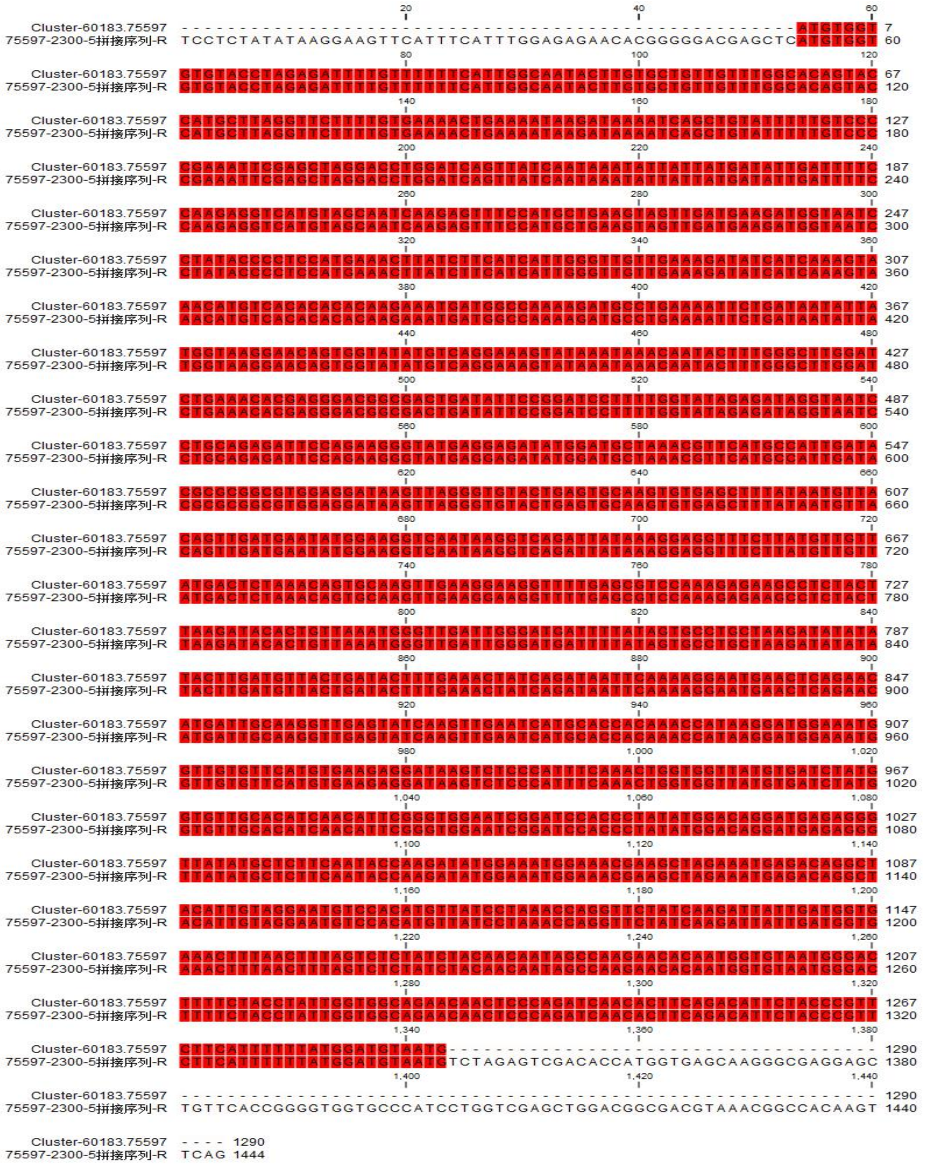

Supplementary Figure 5 Sequencing results of vector (A: pCAMBIA2300-*MrbZIP*-GFP; B: pCAMBIA2300-*MrERF*-GFP; C: pCAMBIA2300-*MrSURNod*-GFP)
