# Supplementary material for: MrERF, MrbZIP, and MrSURNod of Medicago ruthenica Are Involved in Plant Growth and Abiotic Stress Response
Source: Front Plant Sci. 2022 Jun 2;13:907674. doi: 10.3389/fpls.2022.907674 (PMC9203031; doi:10.3389/fpls.2022.907674)
Supplement: Supplementary file 8 [file Table_1.DOCX]

### Supplementary Table S1 Primers for construction of plants overexpression vectors for 3 target genes

| Gene name | Primer (5’ to 3’) | |
| --- | --- | --- |
| *MrERF* | F: AGAACACGGGGGACGAGCTC  ATGGAGAAACATATCAACATGGAAAGTGAT | R: ACCATGGTGTCGACTCTAGA GTATTTCTCCCAAAAAGATGGTTCCTC |
| *MrbZIP* | F: AGAACACGGGGGACGAGCTC ATGAATTTCAAGGGTTTTGGGAATGATC | R: ACCATGGTGTCGACTCTAGA CCATGGACCAGTTTGTGTTCTTCTT |
| *MrSURNod* | F: AGAACACGGGGGACGAGCTC ATGTGGTGTGTACCTAGAGATTTTGTT | R: ACCATGGTGTCGACTCTAGA CATTACATCCATAAAAAATGAAGAACGGG |
| *2300-GFP* | F:ACCGTAAAGCACGAGGAAG | R:GTTGTCACTGAAGCGGGAA |

### Supplementary Table S2 Primers for qRT-PCR

| Gene name | Primer (5’ to 3’) | | Tm |
| --- | --- | --- | --- |
| *MrERF* | F:TTGGCTCACGAATCTCACATACA | R:GCAAAGGACACAAAGGAGAGTAA | 58℃ |
| *MrbZIP* | F:CTTCAACAGCAGCATCAACAACAG | R:ACCATACCAATCCCTCCACCTT | 57℃ |
| *MrSURNod* | F: CGTGGAACAGCGACCGATA | R:ACATTAAGCAACCATTTCTCCTCAA | 55℃ |
| *Actin* | F: CCTGAGGTCCTTTTCCAACCA | R: GGATTCCGGCAGCTTCCATT | ditto |
